# Characterizing inflammatory markers in highly aggressive soft tissue sarcomas

**DOI:** 10.1097/MD.0000000000030688

**Published:** 2022-09-30

**Authors:** Kazuhiko Hashimoto, Shunji Nishimura, Yu Shinyashiki, Tomohiko Ito, Masao Akagi

**Affiliations:** a Department of Orthopedic Surgery, Kushimoto Municipal Hospital, Higashimuro-Gun, Wakayama, Japan; b Department of Orthopedic Surgery, Kindai University Hospital, Osaka-Sayama City, Osaka, Japan.

**Keywords:** blood marker, inflammation, prognostic factor, soft tissue sarcomas

## Abstract

The prognosis for soft tissue sarcomas (STSs) is poor, especially for highly aggressive STSs, and the details of prognostic factors are unknown. This study aimed to investigate the prognostic factors for STSs in hematologic inflammatory markers. We included 22 patients with STSs treated at our institution. The STSs were histologically classified as follows: undifferentiated pleomorphic sarcoma, 7 cases; myxofibrosarcoma, 6 cases; and malignant peripheral nerve sheath tumor, 2 cases. The average patient age was 72.06 years. The numbers of patients who underwent each procedure were as follows: wide resection, 7; wide resection and flap, 2; marginal resection, 2; wide resection and radiation, 1; additional wide resection with flap, 1; wide resection and skin graft, 1; and radiotherapy only, 1. The median follow-up period was 26 months (3–92 months). The outcomes were as follows: continuous disease free, 6 cases; no evidence of disease, 6 cases; alive with disease, 1 case; and died of disease, 2 cases. Pretreatment blood examinations for C-reactive protein (CRP) and albumin levels; neutrophil, lymphocyte, and white blood cell (WBC) counts; and neutrophil/lymphocyte (N/L) ratio were investigated and correlated with tumor size, tissue grade, and maximum standardized uptake value (SUVmax). CRP level and neutrophil and WBC counts were positively correlated with tissue grade and SUVmax. N/L ratio was positively correlated with tumor size and SUVmax. CRP level, WBC and neutrophil counts, and N/L ratio may be poor prognostic factors for highly aggressive STSs.

## 1. Introduction

Soft tissue sarcomas (STSs) are rare neoplasms originating from the malignant transformation of primary, multipotent mesenchymal stem cells.^[1]^ STSs have a wide variety of histological types, accounting for approximately 1% of adult malignancies.^[2]^ The main axis of treatment for STSs is wide resection, and chemotherapy and radiotherapy are useful adjuvant treatments.^[3,4]^ Although pazopanib, trabectedin, and eribulin have been approved for the treatment of high-grade STSs recently, their efficacy is not sufficient.^[5–7]^ However, the prognosis of patients with STSs has reached approximately 65% for 5-year survival, and the prognosis of high-grade STSs or younger patients with STSs is slightly less than that.^[8,9]^ Early diagnosis of STSs is necessary for obtaining favorable outcomes as another factor in the poor prognosis of STSs is delayed diagnosis.^[10,11]^ Magnetic resonance imaging, computed tomography, and positron emission tomography are used to diagnose STSs; however, they are time-consuming and expensive.^[12,13]^

In recent years, specific genetic diagnostics for STSs have been developed, although the types of STSs that can be addressed are limited.^[14]^ Thus, there is a need for a diagnostic marker for STSs that is relatively inexpensive and that can provide early results. Recent studies report the usefulness of blood examinations for inflammatory markers in the diagnosis and prognostic prediction of various malignancies.^[15–17]^ The usefulness of hematologic inflammatory markers in diagnosing and predicting the prognosis of STSs has also been reported in several studies, although evidence for highly aggressive STSs is still lacking.^[18,19]^ Therefore, we conducted a retrospective study to comprehensively search for and characterize blood inflammation markers that may be useful for the diagnosis and prognosis of highly aggressive STSs.

## 2. Methods

The patient characteristics are presented in Table 1. Twenty-two patients with STSs were enrolled in the current study. Finally, 15 cases (in total) (a division of the party: undifferentiated pleomorphic sarcoma [UPS], 7; myxofibrosarcoma [MFS], 6; and malignant peripheral nerve sheath tumor [MPNST], 2 cases) in which 2 or more pretreatment hematologic inflammatory markers were obtained were included. Data on inflammatory markers in preoperative blood test findings were collected as much as possible retrospectively. All patients were treated at our hospital between January 2006 and December 2019. This study was approved by the Kindai University Ethics Committee (approval number: 31-253). Written informed consent was obtained from all patients. Comprehensive consent was obtained from patients who were unable to sign the consent form.

**Table 1:** Characteristics of the study population.

Factor	Patients, n
Age (yr)	
>70	10
≦70	5
Sex	
Male	8
Female	7
Tumor site	
Arms	2
Legs	7
Trunk	6
Histological type	
MFS	6
UPS	7
MPNST	2
Histological grade	
Grade 1	2
Grade 2	5
Grade 3	8
Tumor size	
<5	7
5–10	6
>10	2
SUVmax	
<5	2
5–10	4
>10	3
Treatment	
Wide resection, flap	2
Wide resection, postoperative radiation	1
Wide resection	7
Wide resection, skin graft	1
Additional wide resection	1
Marginal resection	2
Radiation	1
Recurrence	
(+)	6
(−)	9
Metastasis	
(+)	4
(−)	11
Outcome	
CDF	6
NED	6
AWD	1
DOD	2
Follow-up periods (years)	
>3	8
≧3	14

AWD = alive with disease, CDF = continuous disease free, DOD = dead of disease, MFS = myxofibrosarcoma, MPNST = malignant peripheral nerve sheath tumor, NED = no evidence of disease, SUVmax = maximum standardized uptake value, UPS = undifferentiated pleomorphic sarcoma.

### 2.1. Markers in the blood examination

We evaluated each patient’s pretreatment C-reactive protein ([CRP] μL) and albumin (g/dL) levels, as well as white blood cell ([WBC] μL), neutrophil (μL/mm^3^), and lymphocyte (μL) counts, and neutrophil/lymphocyte (N/L) ratio based on historical blood data. The cases in which one or more markers could be collected included MFS, 6 cases; UPS, 7 cases; and MPNST, 2 cases (Table 2). Data could be collected for WBC count, CRP level, albumin level, neutrophil count, lymphocyte count, and N/L ratio in 15, 11, 12, 6, 9, and 5 cases, respectively.

**Table 2:** Inflammatory marker values in each patient.

Patient no.	Histology	WBC (μL)	CRP (mg/L)	Albumin (g/dL)	Neutrophil (μL/mm^3^)	Lymphocyte (μL)	N/L ratio
1	MFS	4000	0.056	4.3	2008	1348	1.48
2	MFS	N/A	N/A	N/A	N/A	N/A	N/A
3	MFS	8700	0.28	3.4	N/A	1853	N/A
4	MFS	7460	1.508	N/A	5296	1641	3.22
5	MFS	7600	N/A	4.2	N/A	N/A	N/A
6	MFS	5270	0.132	N/A	1602	1602	1
7	MFS	N/A	N/A	N/A	N/A	N/A	N/A
8	MFS	6100	0.04	4.6	N/A	1323	N/A
9	MFS	N/A	N/A	N/A	N/A	N/A	N/A
10	UPS	9500	7.2	3.2	7391	N/A	N/A
11	UPS	8200	3.9	4.2	6100	1287	4.73
12	UPS	N/A	N/A	N/A	N/A	N/A	N/A
13	UPS	11810	3.257	3.2	7145	3483	2.05
14	UPS	3200	0.05	4.5	N/A	729	N/A
15	UPS	N/A	N/A	N/A	N/A	N/A	N/A
16	UPS	7300	N/A	3.8	N/A	N/A	N/A
17	UPS	8100	N/A	4.2	N/A	N/A	N/A
18	UPS	5200	N/A	3.6	N/A	N/A	N/A
19	UPS	N/A	N/A	N/A	N/A	N/A	N/A
20	MPNST	3500	0.026	N/A	N/A	N/A	N/A
21	MPNST	N/A	N/A	N/A	N/A	N/A	N/A
22	MPNST	11500	0.37	4.5	N/A	3737	N/A

CRP = C-reactive protein, MFS = myxofibrosarcoma, MPNST = malignant peripheral sheath tumor, N/A = not applicable, N/L = neutrophil/lymphocyte, UPS = undifferentiated pleomorphic sarcoma, WBC = white blood cell.

### 2.2. Correlation between markers in the blood examination and tumor size, histological grade, or maximum standardized uptake value

We investigated the correlation between markers in the blood examination and tumor size, histological grade, or maximum standardized uptake value (SUVmax) value. Data on tumor size and histological grade were available in 22 cases. The SUVmax values could be collected in 13 cases. Tumor size and WBC count (15 cases), CRP level (11 cases), albumin level (12 cases), neutrophil count (6 cases), lymphocyte count (9 cases), and N/L ratio (5 cases) were compared. Histological grade and WBC count (15 cases), CRP level (11 cases), albumin level (12 cases), neutrophil count (6 cases), lymphocyte count (9 cases), and N/L ratio (5 cases) were compared. SUVmax and WBC count (9 cases), CRP level (4 cases), albumin level (8 cases), neutrophil count (4 cases), lymphocyte (6 cases), and N/L ratio (3 cases) were compared.

### 2.3. Comparison of marker values among remission and non-remission cases

The inflammatory marker values in remission cases were compared to those in non-remission cases. WBC counts were compared among 6 remission cases and 9 non-remission cases. CRP levels were compared among 4 remission cases and 7 non-remission cases. Albumin levels were compared among 5 remission cases and 7 non-remission cases. Neutrophil counts were compared among 4 remission cases and 2 non-remission cases. N/L ratios were compared among 3 remission cases and 2 non-remission cases. Lymphocyte counts were compared among 3 remission cases and 6 non-remission cases.

### 2.4. Statistical analysis

The marker values and clinical parameters were plotted. Then, a correlation diagram was constructed.^[21]^ The coefficient of determination (r) was calculated by drawing an approximation line to examine the correlation between each marker. Pearson single liner regression test was used to confirm significant correlations. The *R*-value criteria were as follows: strong, values between 0.7 and 1.0 (–0.7 and –1.0); moderate, values between 0.3 and 0.7 (0.3 and –0.7); weak, values between 0 and 0.3 (0 and –0.3); and no linear relationship, value of 0 as previously described.^[21]^ Student *t* test was used for the statistical analysis and *P* < .05 was considered significant.

## 3. Results

The median age was 72.06 years (range: 34–101 years), and there were 8 men and 7 women. The tumor was located in the upper extremities in 2 cases, the lower extremities in 7 cases, and the trunk in 6 cases. The histological grades were grades 1, 2, and 3 in 2, 5, and 8 patients, respectively.

The median tumor diameter was 5.9 cm (range: 1.5–15.1 cm). The median SUVmax was 10.9 (range: 3.55–15.96). The treatments consisted of wide resection with a flap in 2 cases, wide resection and skin graft in 1 case, additional wide resection with flap in 1 case, wide resection and postoperative radiotherapy in 1 case, wide resection in 7 cases, marginal resection in 2 cases, and postoperative radiotherapy in only 1 case. There were 6 recurrence cases. Moreover, there were 4 cases of metastasis. The final clinical outcomes were continuous disease free in 6, no evidence of disease in 6, alive with disease in 1, and dead of disease in 2 patients. Table 2 summarizes the WBC, CRP, albumin, neutrophil, lymphocyte, and N/L ratio values. The mean (range) value was 6435 (3200–11810) for WBC count, 1.69 (0.026–3.257) for CRP level, 3.6 (3.2–4.6) for albumin level, 4576 (1602–7145) for neutrophil count, 1602 (729–1853) for lymphocyte count, and 1.48 (1–4.73) for N/L ratio.

### 3.1. Correlation between blood markers and tumor size

**Figure 1.**
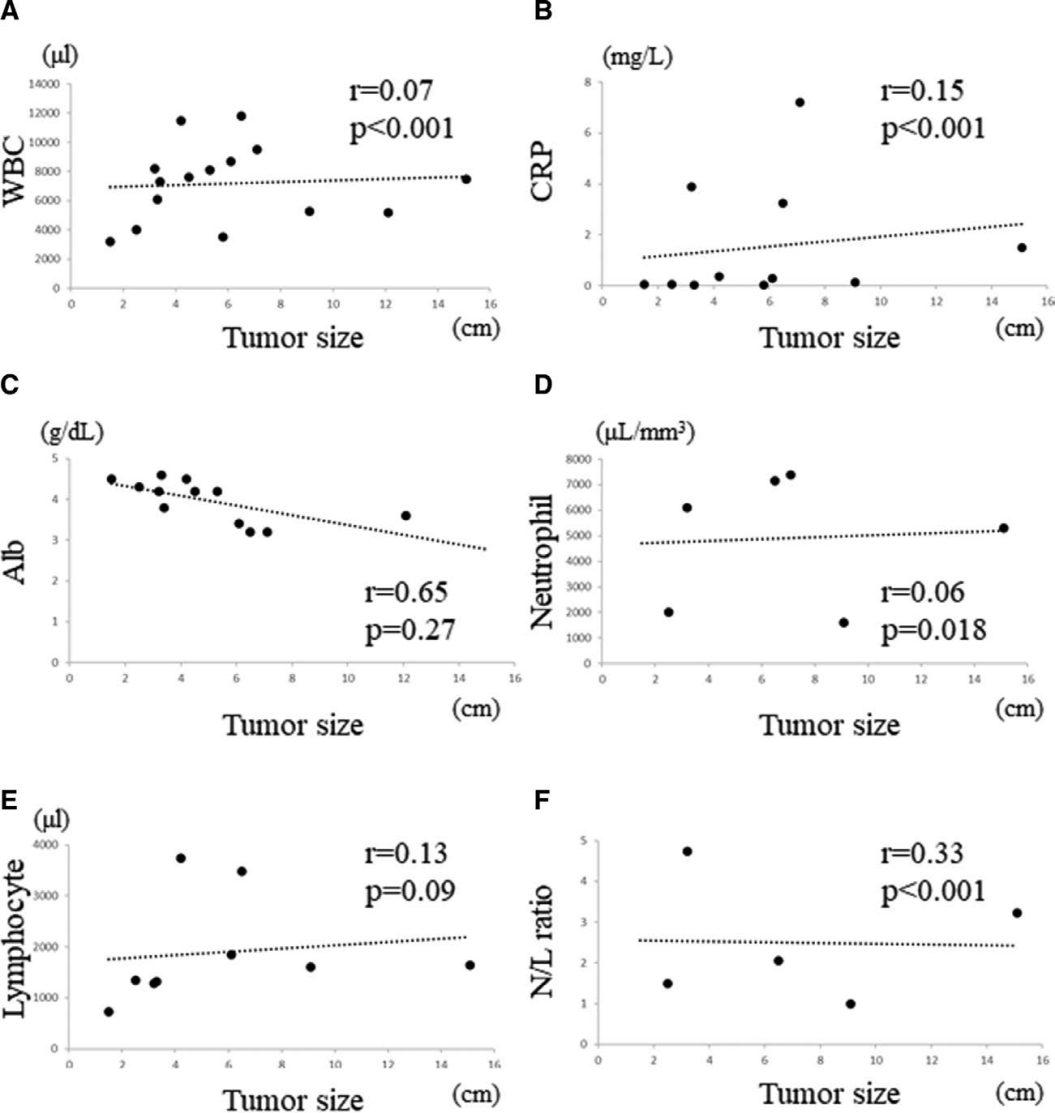
Graphs show no significant correlation between the WBC and tumor size (*R* = 0.07, *P* < .001) in STSs (A); no significant correlation between the CRP level and tumor size (*R* = 0.15, *P* < .001) in STSs (B); no significant correlation between the albumin level and tumor size (*R* = 0.65, *P* = .27) in STSs (C); no significant correlation between the neutrophil and tumor size (*R* = 0.06, *P* = .018) in STSs (D); no positive correlation between the lymphocyte and tumor size (*R* = 0.13, *P* = .09) in STSs (E); no significant correlation between the N/L ratio and tumor size (*R* = 0.33, *P* < .001) in STSs (F). CRP = C-reactive protein, N/L = neutrophil/lymphocyte, STSs = soft tissue sarcomas, WBC = white blood cell.

No significant correlation was observed between WBC count and tumor size (*R* = 0.07, *P* < .001, Fig. 1A) or between CRP level and tumor size (*R* = 0.15, *P* < .001, Fig. 1B). No significant correlation was observed between albumin level and tumor size (*R* = 0.65, *P* = .27, Fig. 1C), between neutrophil count and tumor size (*R* = 0.06, *P* = .018, Fig. 1D), or between lymphocyte count and tumor size (*R* = 0.13, *P* = .09, Fig. 1E). Furthermore, no correlation was observed between N/L ratio and tumor size (*R* = 0.33, *P* < .001, Fig. 1F).

### 3.2. Correlation between blood markers and histological grade

**Figure 2.**
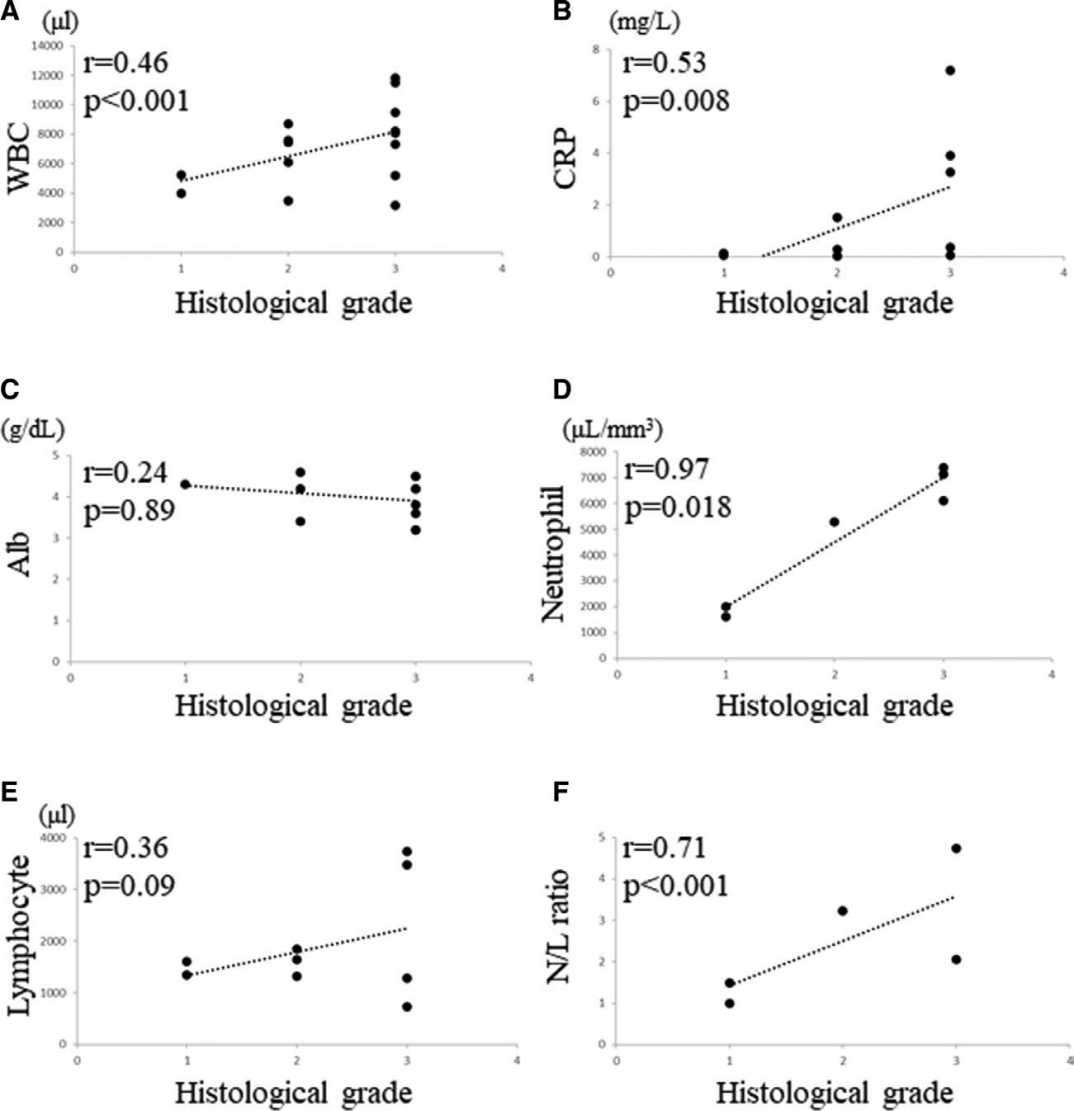
Graphs show no significant correlation between the WBC and histological grade (*R* = 0.46, *P* < .001) in STSs (A); no significant correlation between the CRP and histological grade (*R* = 0.53, *P* = .008) in STSs (B); no significant correlation between the albumin and histological grade (*R* = 0.24, *P* = .89) in STSs (C); no significant correlation between the neutrophil count and histological grade (*R* = 0.97, *P* = .018) in STSs (D); no positive correlation between the lymphocyte count and histological grade (*R* = 0.36, *P* = .09) in STSs (E); no significant correlation between the N/L ratio and histological grade (*R* = 0.71, *P* < .001) in STSs (F). CRP = C-reactive protein, N/L = neutrophil/lymphocyte, STSs = soft tissue sarcomas, WBC = white blood cell.

The correlation between WBC count and histological grade was significantly moderately positive (*R* = 0.46, *P* < .001, Fig. 2A), and a significant strong correlation was observed between CRP level and histological grade (*R* = 0.53, *P* = .008, Fig. 2B). There was no significant correlation between albumin level and histological grade (*R* = 0.24, *P* = .89, Fig. 2C); however, we observed a significant strong correlation between neutrophil count and histological grade (*R* = 0.97, *P* = .018, Fig. 2D). There was no significant correlation between lymphocyte count and histological grade (*R* = 0.36, *P* = .09, Fig. 2E); however, we observed a significant strong correlation between N/L ratio and histological grade (*R* = 0.71, *P* < .001, Fig. 2F).

### 3.3. Correlation between blood markers and maximum standardized uptake value

**Figure 3.**
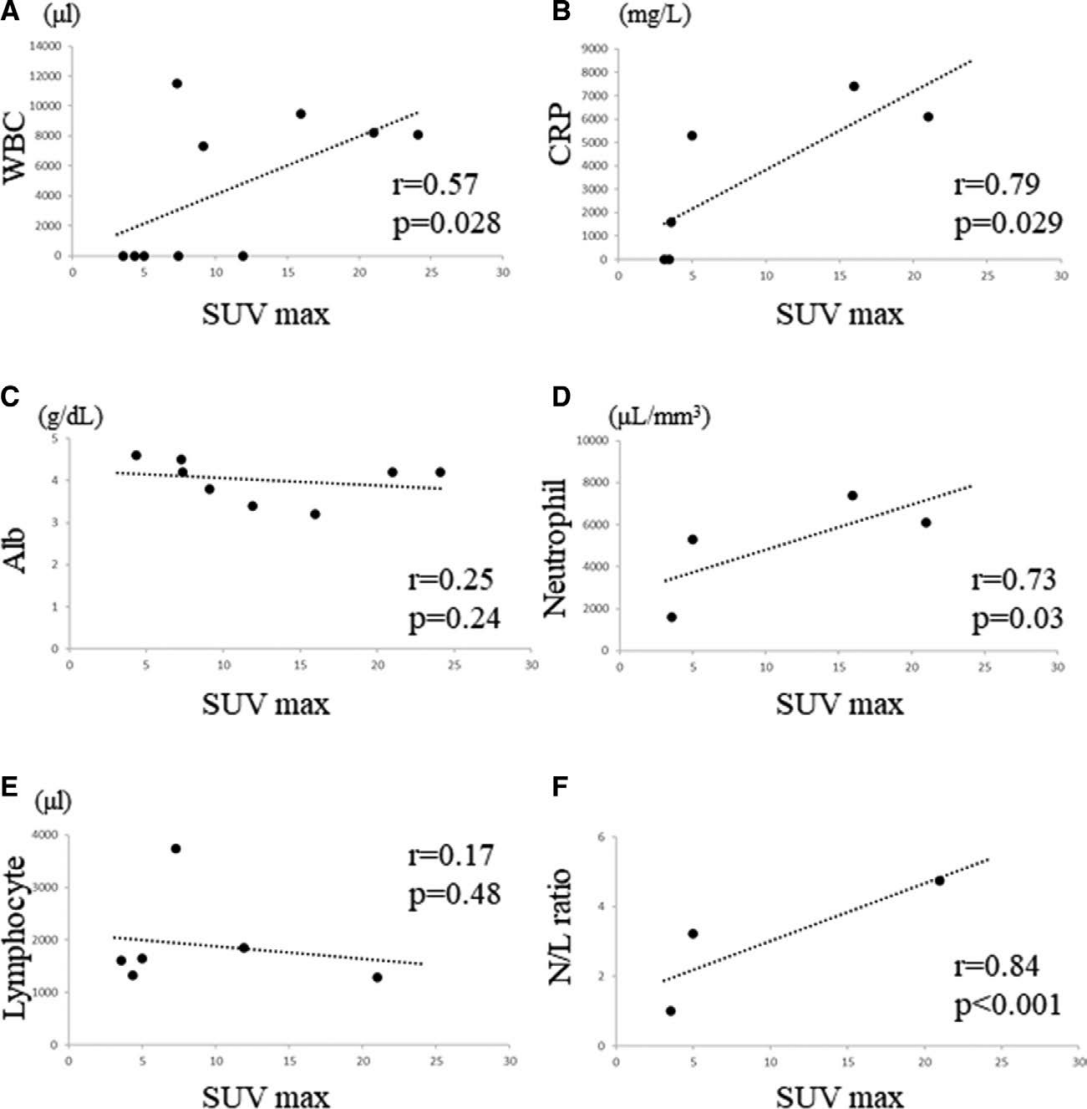
Graphs show no significant correlation between the WBC count and SUVmax (*R* = 0.57, *P* = .028) in STSs (A); no significant correlation between the CRP level and SUVmax (*R* = 0.79, *P* = .029) in STSs (B); no significant correlation between the albumin level and SUVmax (*R* = 0.25, *P* = .24) in STSs (C); significant strong correlation between the neutrophil count and SUVmax (*R* = 0.73, *P* = .03) in STSs (D); no positive correlation between the lymphocyte count and SUVmax (*R* = 0.17, *P* = .48) in STSs (E); significant strong correlation between the N/L ratio and SUVmax (*R* = 0.84, *P* < .001) in STSs (F). CRP = C-reactive protein, N/L = neutrophil/lymphocyte, STSs = soft tissue sarcomas, SUVmax = maximum standardized uptake value, WBC = white blood cell.

The correlation between WBC count and the SUVmax was significantly moderately positive (*R* = 0.57, *P* = .0028, Fig. 3A). A significant strong correlation between CRP level and SUV max was observed (*R* = 0.79, *P* = .029, Fig. 3B); however, there was no significant correlation between albumin level and SUVmax (*R* = 0.25, *P* = .24, Fig. 3C). We observed a significant strong correlation between neutrophil count and SUVmax (*R* = 0.73, *P* = .03, Fig. 3D), although there was no significant correlation between lymphocyte count and SUVmax (*R* = 0.17, *P* = .48, Fig. 3E). Moreover, we observed a significant strong correlation between N/L ratio and SUVmax (*R* = 0.84, *P* < .001, Fig. 3F).

### 3.4. Differences in inflammatory markers between remission cases (continuous disease free) and non-remission cases (no evidence of disease, alive with disease, and dead of disease)

Table 3 summarizes the WBC, CRP, albumin, neutrophil, lymphocyte, and N/L ratio values of remission and non-remission cases.

**Table 3:** CRP, WBC, albumin, neutrophil, lymphocyte, and N/L ratio values in continuously disease-free or no-evidence-of-disease cases compared to alive with disease and died of other cause cases.

Marker	CRP		WBC		Albumin		Neutrophil		Lymphocyte		N/L ratio	
Outcome	CDF or NED	AWD or DOD	CDF or NED	AWD or DOD	CDF or NED	AWD or DOD	CDF or NED	AWD or DOD	CDF or NED	AWD or DOD	CDF or NED	AWD or DOD
Mean	1.69	0.28	6435	7460	3.6	4.2	4576	5698	1602	1482	1.48	3.97
SD	3.37	1.42	4093	2585	0.52	0.43	3160	569	1166	1040	0.52	1.06
*P* value	.24		.75		.12		.65		.63		.03	

AWD = alive with disease, CDF = continuous disease free, CRP = C-reactive protein, DOD = dead of disease, N/L = neutrophil/lymphocyte, NED = no evidence of disease, SD = standard deviation, WBC = white blood cell.

The WBC count in remission cases was 6435 ± 4093 (average ± standard deviation [SD]) and that of non-remission cases was 5698 ± 2585 (average ± SD). There was no significant difference between the WBC count of remission cases and that of non-remission cases (*P* = .75).

The CRP level in remission cases was 1.69 ± 3.37 (average ± SD) and that of non-remission cases was 0.28 ± 1.42 (average ± SD). There was no significant difference between the CRP level of remission cases and that of non-remission cases (*P* = .24).

The albumin level in remission cases was 3.6 ± 0.52 (average ± SD) and that of non-remission cases was 4.2 ± 0.43 (average ± SD). There was no significant difference between albumin levels of remission cases and those of non-remission cases (*P* = .12).

The neutrophil count in remission cases was 4576 ± 3160 (average ± SD) and that of non-remission cases was 5698 ± 568 (average ± SD). There was no significant difference between neutrophil counts of remission cases and those of non-remission cases (*P* = .65).

The lymphocyte count in remission cases was 1602 ± 1166 (average ± SD) and that of non-remission cases was 1482 ± 1040 (average ± SD). There was no significant difference between lymphocyte counts of remission cases and those of non-remission cases (*P* = .63).

The N/L ratio in remission cases was 1.45 ± 0.52 (average ± SD) and that of non-remission cases was 3.97 ± 1.06 (average ± SD). The N/L ratio in non-remission cases was significantly larger than that in remission cases (*P* = .03).

## 4. Discussion

Chronic inflammation plays an important overall role in the pathogenesis, treatment, and prognosis of STS as previously described.^[22]^ We investigated the prognostic inflammatory blood markers for highly aggressive STS.

A previous study reported that STS patients with elevated CRP levels prior to treatment had lower disease-specific survival rates,^[18]^ while another revealed that high preoperative CRP levels significantly increased the risk of STS recurrence and decreased overall survival.^[23]^ Moreover, elevated preoperative CRP level is reportedly an independent prognostic factor for predicting poor prognosis in STS patients.^[24]^ In the current study, CRP levels had significant positive correlations with poor prognostic factors for STS such as tumor size, histological grade, and SUVmax.^[25–28]^ These findings suggest that preoperative CRP level may be a poor prognostic factor for highly aggressive STSs.

A previous study suggested that the preoperative CRP/albumin ratio is an independent prognostic factor for STS that shows superior prognostic ability compared to established inflammation-based prognostic indicators.^[29]^ In addition, in vivo studies have shown decreased albumin/globulin ratios in patients with malignant STSs compared to benign STSs, and it has been reported that pretreatment albumin/globulin ratios may support diagnosis and optimal treatment planning.^[30]^ No significant correlations between preoperative albumin level and poor prognostic factors for STS such as tumor size, histological grade, and SUVmax^[26–29]^ were observed in the present study. Therefore, albumin level may not be a poor prognostic factor for highly aggressive STSs.

Pretreatment N/L ratio is reportedly a potential biomarker for poor prognosis in STSs.^[31]^ In addition, a previous study has shown that preoperative lymphocyte/monocyte ratio is a new independent prognostic factor predicting clinical outcomes in STS patients.^[32]^ Interestingly, in adult patients with STSs, a combination of pretreatment CRP levels and N/L ratios can reportedly predict disease-specific survival.^[18]^ In the current study, WBC and neutrophil counts had significant positive correlations with poor prognostic factors for STS such as histological grade and SUVmax.^[25–28]^ Furthermore, significant correlations were observed between N/L ratio and tumor size, histological grade, and SUVmax. Therefore, WBC count, neutrophil count, and N/L ratio may be a poor prognostic factor for highly aggressive STSs.

In the current study, we could not observe significant differences between the inflammatory markers of remission cases and those of non-remission cases, possibly due to the small sample size. Moreover, we considered that the unique tumor microenvironment in highly aggressive STSs may be different from that in less aggressive STSs.

## 5. Limitations

The current study had some limitations. First, the study included a small cohort. Second, the treatment modalities were different in each case. However, based on the guidelines of each era, standard treatment methods were implemented. Third, we could not determine a significant difference in inflammatory marker values between remission and non-remission cases. Nevertheless, we have confirmed the involvement of inflammatory markers in the prognosis of highly aggressive STSs. The strength of this study is that it is the first to examine the significance of inflammatory markers in highly progressive STSs. Further studies with larger sample sizes and long-term clinical follow-up durations are warranted to clarify that inflammatory markers are significantly involved in the prognosis of highly progressive STSs.

CRP level, WBC count, neutrophil count, and N/L ratio may be poor prognostic factors for patients with UPS, MFS, and MPNST.

## Acknowledgment

The authors thank Editage for the English language editing.

## Author contributions

**Conceptualization:** Kazuhiko Hashimoto, Tomohiko Ito, Masao Akagi.

**Data curation:** Kazuhiko Hashimoto, Shunji Nishimura, Yu Shinyashiki, Tomohiko Ito.

**Formal analysis:** Kazuhiko Hashimoto, Shunji Nishimura, Yu Shinyashiki, Masao Akagi.

**Investigation:** Kazuhiko Hashimoto, Shunji Nishimura, Yu Shinyashiki, Tomohiko Ito.

**Methodology:** Kazuhiko Hashimoto, Shunji Nishimura, Yu Shinyashiki, Tomohiko Ito, Masao Akagi.

**Project administration:** Kazuhiko Hashimoto, Shunji Nishimura, Masao Akagi.

**Resources:** Kazuhiko Hashimoto, Shunji Nishimura.

**Software:** Kazuhiko Hashimoto, Tomohiko Ito, Masao Akagi.

**Supervision:** Kazuhiko Hashimoto, Shunji Nishimura, Masao Akagi.

**Validation:** Kazuhiko Hashimoto, Shunji Nishimura, Yu Shinyashiki, Tomohiko Ito, Masao Akagi.

**Visualization:** Kazuhiko Hashimoto, Yu Shinyashiki.

**Writing – original draft:** Kazuhiko Hashimoto, Shunji Nishimura, Yu Shinyashiki, Tomohiko Ito, Masao Akagi.

**Writing – review & editing:** Kazuhiko Hashimoto, Shunji Nishimura, Yu Shinyashiki, Tomohiko Ito, Masao Akagi.
